# Supplementary material for: Content-rich biological network constructed by mining PubMed abstracts
Source: BMC Bioinformatics. 2004 Oct 8;5:147. doi: 10.1186/1471-2105-5-147 (PMC528731; doi:10.1186/1471-2105-5-147)
Supplement: Additional File 2 — The original results of the above study (non-essential files are deleted to keep the file size under the limit set by BMC bioinformatics). [file 1471-2105-5-147-S2.bz2 › chilibotAdditionalFile2/dip50/348ID7721786E4496/html/IL4R_IL13.html]

 


 **IL4R** and **IL13** 
  
Found 306 abstracts in PubMed, retrieved 50.  
 

 What does Google say? 
 PDF only 
| .edu only 

---

**Interactive relationship** (e.g. stimulation, inhibition, etc)

**Neutral relationship**- Association and Interaction of the  **IL4R** , IL4, and  **IL13**  Loci with Type 1 Diabetes among Filipinos.  Ref: 12748907 Am J Hum Genet, 2003
- Interleukin 4  [ **IL4R** ]  IL 4 and IL 13  [ **IL13** ]  are the only cytokines known to bind to the receptor chain IL 4Ralpha.  Ref: 12421669 Biochim Biophys Acta, 2002

**Non-interactive relationship** (e.g. studied together, co-existance, homology, etc.)

- Supernatants were assayed for the cytokines interleukin 4  [ **IL4R** ]  IL 4, granulocyte macrophage colony stimulating factor GM CSF, IL 6, IL 8, IL 13  [ **IL13** ]  and monocyte chemotactic protein 1 MCP 1.  Ref: 12456364 Front Biosci, 2003
- Furthermore, both interleukin 4  [ **IL4R** ]  IL 4 and IL 13  [ **IL13** ] , but not a range of cytokines induce expression of the variant in bronchial epithelial cells.  Ref: 12137938 Gene, 2002
- It is known that both interleukin 4  [ **IL4R** ]  IL 4 and IL 13  [ **IL13** ]  are produced by Th2 type cells and share similar biological functions with each other.  Ref: 12441140 Cytokine, 2002
- Our data suggest that the risk for T1D is determined, in part, by polymorphisms within the  **IL4R**  locus, including promoter and coding sequence variants, and by specific combinations of genotypes at the  **IL4R**  and the IL4 and  **IL13**  loci.  Ref: 12748907 Am J Hum Genet, 2003
- We studied the effects of interferon gamma IFN gamma, a Th1 cytokine, and interleukin 13  [ **IL13** ]  IL 13  [ **IL13** ]  or interleukin 4  [ **IL4R** ]  IL 4, Th2 cytokines, on the antifungal activity of resident murine peritoneal macrophages against Candida albicans in vitro.  Ref: 12003965 J Antimicrob Chemother, 2002
- Interleukin 4  [ **IL4R** ]  IL 4 and IL 13  [ **IL13** ]  are two cytokines produced by T helper type 2 cells, mast cells, and basophils.  Ref: 12791978 Science, 2003
- We determined the allele frequencies of five polymorphisms in the  **IL13** , IL13RA1, and IL4RA  [ **IL4R** ]  genes in 588 continuing smokers chosen from the NHLBI Lung Health Study for having the fastest n = 282 and slowest n = 306 5 yr rate of decline of lung function mean change in FEV 1 %predicted yr = 4.1 and 1.1, respectively.  Ref: 12594065 Am J Respir Cell Mol Biol, 2003
- Here we show that mice lacking interleukin 12p35 IL 12p35 following T. crassiceps infection, failed to mount a Th1 response, but developed a strong Th2 type response, produced higher levels of IgG1, IgE, interleukin 4  [ **IL4R** ] , interleukin 5 as well as interleukin 13  [ **IL13** ]  than wild type mice, and became highly susceptible to the larval stage of this cestode.  Ref: 12814650 Int J Parasitol< MedlineTA>Int J Parasitol, 2003
- Although there were no significant differences in the levels of interleukin 4  [ **IL4R** ] , IL 5, IL 13  [ **IL13** ] , eotaxin, granulocyte macrophage colony stimulating factor, RANTES, or eosinophil cationic protein, there was a marked increase in eosinophil derived neurotoxin both systemically and in the lungs of individuals with TPE compared to each of the control groups.  Ref: 12595450 Infect Immun, 2003
- Type I IgE receptor, interleukin 4 receptor  [ **IL4R** ]  and interleukin 13  [ **IL13** ]  polymorphisms in children with nephrotic syndrome.  Ref: 11980568 Clin Sci Lond , 2002
- This effect is likely the result of the elaboration of T2 cytokine levels, because pulmonary instillation of interleukin 4  [ **IL4R** ]  or interleukin 13  [ **IL13** ]  induces the accumulation of Ear11 transcripts in naive animals.  Ref: 12444027 Am J Respir Cell Mol Biol, 2002
- Among the cytokines inhibited were interleukin 4  [ **IL4R** ]  IL 4, IL 13  [ **IL13** ] , IFNgamma, tumour necrosis factor alpha TNFalpha and granulocyte macrophage colony stimulating factor.  Ref: 12777636 Rheumatology Oxford < MedlineTA>Rheumatology Oxford , 2002
- We hypothesized that variants in the  **IL13** , IL13RA1, and IL4RA  [ **IL4R** ]  genes would be associated with an accelerated rate of decline of lung function among smokers.  Ref: 12594065 Am J Respir Cell Mol Biol, 2003
- The roles of interleukin 4  [ **IL4R** ]  IL 4 and IL 13  [ **IL13** ]  in the regulation of immunity to Leishmania donovani infection are still poorly understood.  Ref: 12874364 Infect ImmunInfect Immun, 2003
- In order to compare the regulatory profiles of TARC production by tumor necrosis factor alpha TNF alpha, IFN gamma, interleukin 4  [ **IL4R** ]  IL 4 and IL 13  [ **IL13** ]  in keratinocytes and fibroblasts, HaCaT cells, a human keratinocyte cell line, and NG1RGB cells, a human skin fibroblast cell line, were used.  Ref: 12354417 J Dermatol Sci, 2002
- Both interleukin 4  [ **IL4R** ]  IL 4 and IL 13  [ **IL13** ]  inhibited TNF alpha and IFN gamma enhanced MDC production in HaCaT cells in a dose dependent manner.  Ref: 12670721 J Dermatol Sci, 2003
- Furthermore, cysteine proteinases induced neither interleukin 4  [ **IL4R** ]  IL 4 nor IL 13  [ **IL13** ]  and low levels of IL 10 in controls and patients.  Ref: 12704171 Infect Immun, 2003
- Asthma is mediated by Th2 lymphocytes, which produce a limited repertoire of cytokines, including interleukin 4  [ **IL4R** ]  IL 4, IL 5, IL 9 and IL 13  [ **IL13** ] .  Ref: 12091879 Nat Med, 2002
- Interleukin 4  [ **IL4R** ]  IL 4 and IL 13  [ **IL13** ]  signaling did not influence the susceptibility of BALB c mice to T. congolense infection and interestingly were not the main trigger to alternative macrophage activation.  Ref: 12379696 Infect Immun, 2002
- In contrast, similarly infected CD40 deficient BALB c mice CD40 displayed impairment of both Th1 and Th2 type responses associated with low levels of interferon gamma as well as IgE, interleukin 4  [ **IL4R** ] , interleukin 5 and interleukin 13  [ **IL13** ] , but efficiently controlled T. crassiceps infection.  Ref: 12814650 Int J Parasitol< MedlineTA>Int J Parasitol, 2002
- By reverse transcription polymerase chain reaction, we confirmed an overexpression of the alpha1 chain of the IL 13  [ **IL13** ]  receptor and alpha chain of the interleukin 4 receptor  [ **IL4R** ]  mRNA in lesional skin psoriatic biopsies, when compared with skin biopsies from healthy subjects p< ; 0.01.  Ref: 12445201 J Invest Dermatol, 2002
- Ten single nucleotide polymorphisms SNPs, including two promoter SNPs in the  **IL4R**  locus on chromosome 16p11, one promoter SNP in the IL4 locus on chromosome 5q31, and four SNPs including two promoter SNPs in the  **IL13**  locus on chromosome 5q31 were examined for association, linkage disequilibrium, and interaction.  Ref: 12748907 Am J Hum Genet, 2003
- Characterization of hematopoietic cells also revealed that stimulation of monocytes with interleukin 4  [ **IL4R** ]  IL 4 or IL 13  [ **IL13** ]  was sufficient to induce expression of DC SIGN.  Ref: 12021323 J Virol, 2002
- Dichotomic nature of atopic dermatitis reflected by combined analysis of monocyte immunophenotyping and single nucleotide polymorphisms of the interleukin 4  [ **IL4R** ]  interleukin 13  [ **IL13** ]  receptor gene the dichotomy of extrinsic and intrinsic atopic dermatitis.  Ref: 12406333 J Invest Dermatol, 2002
- The most recent experimental observations suggest that a critical role in the modulation of immune response is sustained by Th2 cytokines, such as interleukin 4  [ **IL4R** ]  IL 4, IL 5 and IL 13  [ **IL13** ] , by co stimulatory molecules, such as CD40 on B cells, and by hormones and neuropeptides.  Ref: 12787302 Pediatr Allergy Immunol, 2003
- Histamine enhances the secretion of Th2 cytokines such as IL 4 interleukin 4  [ **IL4R** ] , IL 5, IL 10 and IL 13  [ **IL13** ]  and inhibits the production of Th1 cytokines IL 2 and IFNgamma interferon gamma and monokine IL 12.  Ref: 12810348 Int Immunopharmacol, 2003
- Interleukin 4  [ **IL4R** ]  and interleukin 13  [ **IL13** ]  signaling connections maps.  Ref: 12791978 Science, 2003
- BACKGROUND Signal transducer and activator of transcription 6 STAT6 is a key transcription factor involved in both interleukin 4  [ **IL4R** ]  IL 4 and IL 13  [ **IL13** ]  mediated biological responses, such as allergies.  Ref: 12759487 Int Arch Allergy Immunol, 2003
- Since IL4 and  **IL13**  both serve as ligands for a receptor composed, in part, of the  **IL4R**  alpha chain, we looked for potential epistasis between polymorphisms in the  **IL4R**  locus on chromosome 16p11 and the five SNPs in the IL4 and  **IL13**  loci on chromosome 5q31 and found, through use of a logistic regression model, significant gene gene interactions P=.045, corrected for multiple comparisons by permutation analysis.  Ref: 12748907 Am J Hum Genet, 2003
- Interleukin 4  [ **IL4R** ] , IL 6, IL 10, IL 11 and IL 13  [ **IL13** ]  were added to cultures for 14 days.  Ref: 12516641 Am J Reprod Immunol, 2002
- Polymorphisms in the  **IL13** , IL13RA1, and IL4RA  [ **IL4R** ]  genes and rate of decline in lung function in smokers.  Ref: 12594065 Am J Respir Cell Mol Biol, 2003
- Interleukin 13  [ **IL13** ]  and interleukin 4  [ **IL4R** ]  were not detected either as mRNA or as the proteins in any of the biopsies from psoriatic patients or healthy subjects.  Ref: 12445201 J Invest Dermatol, 2002
- In vitro expression of interleukin 4  [ **IL4R** ]  IL 4 and IL 13  [ **IL13** ]  mRNA by overnight concanavalin A ConA stimulated draining lymph node cells was enhanced following in vivo treatment with TMA but not with DNCB in the B6C3F1, C57BL 6 and BDF1 mice.  Ref: 12424743 J Appl Toxicol< MedlineTA>J Appl Toxicol, 2002
- Using an in vitro cell culture system, we investigated how the key regulatory cytokines interleukin 4  [ **IL4R** ]  IL 4, IL 10, IL 13  [ **IL13** ] , and TGF beta might modulate the effect of IFN gamma in inducing resistance to infection in enterocyte cell lines.  Ref: 12874337 Infect Immun, 2003
- Neutralizing antibodies against human RANTES, MIP 1alpha, MIP 1beta, alpha interferon IFN alpha, IFN beta, IFN gamma, interleukin 4  [ **IL4R** ]  IL 4, IL 10, IL 13  [ **IL13** ] , IL 16, MCP 1, MCP 3, tumor necrosis factor alpha TNF alpha, or TNF beta failed to reverse the HIV 1 suppressive activity.  Ref: 12885891 J VirolJ Virol, 2003
- Signal transducer and activator of transcription 6 STAT6 is a key transcription factor involved in interleukin 4  [ **IL4R** ]  IL 4 and IL 13  [ **IL13** ]  mediated Th2 response.  Ref: 12605697 Clin Exp Immunol, 2003
- Changes of plasma interleukin 4  [ **IL4R** ] , interleukin 10 and interleukin 13  [ **IL13** ]  in patients with acute respiratory distress syndrome < ArticleTitle>.  Ref: 12490119 Zhonghua Jie He He Hu Xi Za Zhi, 2002
- The effect of disodium cromoglycate, budesonide, and cyclosporin A on interleukin 4  [ **IL4R** ] , interleukin 5, and interleukin 13  [ **IL13** ]  secretions in Der p I stimulated T cells from house dust mite sensitive atopic and nonatopic individuals.  Ref: 12001789 Allergy Asthma Proc< MedlineTA>Allergy Asthma Proc, 2002
- Throughout the course of infection, Taenia crassiceps soluble antigen stimulated spleen cells from both MIF and MIF mice produced significant and comparable levels of interleukin 4  [ **IL4R** ]  IL 4, but those from MIF mice produced significantly more IL 13  [ **IL13** ] , as well as gamma interferon IFN gamma, suggesting that the susceptibility of MIF mice to T. crassiceps was not due to the lack of IFN gamma production.  Ref: 12595439 Infect Immun, 2003
- TSLP activated DCs primed naïve T H cells to produce the proallergic cytokines interleukin 4  [ **IL4R** ]  IL 4, IL 5, IL 13  [ **IL13** ]  and tumor necrosis factor alpha, while down regulating IL 10 and interferon gamma.  Ref: 12055625 Nat Immunol, 2002
- Haplotypes consisting of alleles from the  **IL13**  polymorphisms or from the IL4RA  [ **IL4R** ]  polymorphisms were not associated with rate of decline in lung function in smokers.  Ref: 12594065 Am J Respir Cell Mol Biol, 2003
- The concept of an alternative pathway of macrophage activation by the T H 2 type cytokines interleukin 4  [ **IL4R** ]  IL 4 and IL 13  [ **IL13** ]  has gained credence in the past decade, to account for a distinctive macrophage phenotype that is consistent with a different role in humoral immunity and repair.  Ref: 12511873 Nat Rev Immunol, 2003
- When either mouse interleukin 4  [ **IL4R** ]  or interleukin 13  [ **IL13** ]  was added to calvariae treated with IL 11, Ca release was inhibited.  Ref: 12110441 Bone, 2002
- OBJECTIVE To explore the anti inflammatory mechanisms of interleukin 4  [ **IL4R** ]  IL 4, interleukin 10 IL 10 and interleukin 13  [ **IL13** ]  IL 13  [ **IL13** ]  in acute respiratory distress syndrome ARDS.  Ref: 12490119 Zhonghua Jie He He Hu Xi Za Zhi, 2002
- Interleukin 4  [ **IL4R** ]  and IL 13  [ **IL13** ]  activated phosphorylation of mitogen activated protein kinase in LS174T cells.  Ref: 12848848 Immunol Cell Biol, 2003
- Mice incapable of generating an efficient Th2 response because of functional deletion of the genes for signal transducer and activation of transcription 6 Stat6, interleukin 4 receptor alpha chain  [ **IL4R** ]  IL 4Ralpha, or IL 4 plus IL 13  [ **IL13** ]  IL 4 IL 13  [ **IL13** ]  were no more resistant than wild type WT mice to airborne infection with virulent Mycobacterium tuberculosis.  Ref: 12379724 Infect Immun, 2002
- Polymorphisms in the genes encoding the high affinity IgE receptor, the interleukin 4  [ **IL4R** ]  IL4 receptor and  **IL13**  can be associated with the development of asthma and allergy.  Ref: 11980568 Clin Sci Lond , 2002
- Increased expression of interleukin 13  [ **IL13** ]  but not interleukin 4  [ **IL4R** ]  in CD4 cells from patients with the hyper IgE syndrome.  Ref: 12067309 Clin Exp Immunol, 2002
- Human fetal lung fibroblasts, human adult bronchial fibroblasts and human airway smooth muscle cells were cast into native type I collagen gels and allowed to contract in the presence or absence of IL interleukin 4  [ **IL4R** ] , IL 5, IL 10, or IL 13  [ **IL13** ] .  Ref: 11943670 Am J Physiol Lung Cell Mol Physiol, 2002
